# Robocasting as an Additive Manufacturing Method for Oxide Ceramics: A Study of Mechanical Properties and Microstructure

**DOI:** 10.3390/ma18204775

**Published:** 2025-10-18

**Authors:** Szymon Przybyła, Maciej Kwiatkowski, Michał Kwiatkowski, Marek Hebda

**Affiliations:** 1 Faculty of Materials Engineering and Physics, Cracow University of Technology, Warszawska 24, 31-155 Kraków, Poland; szymon.przybyla@doktorant.pk.edu.pl; 2 Createc Sp. z o.o., E. Kwiatkowskiego 9, 37-450 Stalowa Wola, Poland; maciej.kwiatkowski@createc.com.pl (M.K.); m.kwiatkowski@createc.com.pl (M.K.)

**Keywords:** 3D printing, robocasting, oxide ceramics, mechanical properties, additive manufacturing, direct ink writing, ceramic filters

## Abstract

Additive manufacturing methods can constitute a valuable alternative to conventional production techniques for components used in the heavy industry, particularly in foundry applications. This innovative manufacturing approach enables an expanded product portfolio as well as higher precision and geometrical complexity of ceramic components. One additive technology applicable to ceramic processing is robocasting, classified within the direct ink writing (DIW) family. In this method, a semi-fluid ceramic paste is extruded to build the part layer by layer; the shaped green body is subsequently fired (sintered) to attain its final functional properties. This study presents the results of materials characterization of printed ceramic filters, encompassing phase composition analysis, density measurements, three-point bending strength testing, hardness, and microstructural examination. The investigations demonstrated that the oxide ceramic Al_2_O_3_ processed by the modern robocasting method exhibits mechanical performance at a comparably high level relative to classical manufacturing routes (slip casting, ceramic injection molding, dry pressing). Moreover, the porosity results indicate that 3D printing technology enables lower post-sintering porosity.

## 1. Introduction

Technical ceramics constitute an important area of research in materials engineering. Their unique physical and mechanical properties—such as high resistance to elevated temperatures [1,2,3], wear resistance [4,5,6], insulating performance [7,8,9], and high mechanical strength [10,11,12]—predispose them to use under particularly demanding service conditions. A broad range of industries, including the electrotechnical sector [13,14,15], power generation [16,17,18], machinery and tooling [19,20,21], chemical processing [22,23,24], medical applications [25,26,27], as well as the aerospace [2,28,29] and automotive sectors [30,31,32], exploit the potential of technical ceramics to improve the efficiency and reliability of technological processes. The literature provides numerous studies on the physicochemical properties of oxide ceramics [33,34,35,36]. Of particular significance within this group is alumina (Al_2_O_3_), which—owing to its low manufacturing cost and favorable service characteristics—is among the most widely used ceramic materials [37]. Other ceramic materials include zirconia (ZrO_2_, often stabilized with Y_2_O_3_), silica (SiO_2_), and titania (TiO_2_). Due to their higher cost, these materials are applied mainly as dopants (especially ZrO_2_) or in specialized production.

In response to the dynamic development of technology and the corresponding demand for increasingly complex components, both academia and industry are actively seeking new methods for processing oxide ceramics. Conventional, widely adopted manufacturing techniques—such as uniaxial pressing [38], dip casting [39], or extrusion [40]—do not always permit the fabrication of parts with intricate geometries. One modern direction is additive manufacturing (AM), which has gained prominence in recent years by offering unprecedented capabilities for the design and fabrication of complex ceramic architectures. The most advanced AM methods for ceramics include direct ink writing (DIW) [41,42,43], binder jetting (BJ) [41,44], and stereolithography (SLA) [45]. The diversity of 3D-printing technologies arises primarily from the need to tailor the printed part to its intended application, because these methods are not equivalent in terms of achievable accuracy and mechanical performance. DIW methods are characterized by the ease and speed of paste formulation (both chemically and structurally—e.g., controlled porosity), relatively high green packing leading to >95% of theoretical density after sintering, and low equipment cost [46,47,48]. The drawbacks of DIW include limited printing resolution (typically 100–300 µm), rheology-related challenges, a high risk of cracking during drying, and significant sintering shrinkage [41,49,50]. In turn, BJ is a low-energy process (reduced thermal stresses) and enables the fabrication of geometrically complex structures [51,52,53]. However, it precludes achieving high green density; printed surfaces are comparatively rough—limiting their use in mechanically loaded components—and the resolution is constrained by the particle size of the ceramic powder [51,54,55]. SLA-based 3D printing provides very high precision (25–50 µm), high density (up to 99.9% TD), and allows the creation of complex internal architectures [56,57]. It does, however, require high-quality, chemically stable suspensions that are difficult to formulate, extensive support structures that are challenging to remove, and the process exhibits considerable linear shrinkage that must be compensated for already at the CAD design stage [58,59,60].

Robocasting [61], a DIW-class technique applied to oxide-ceramic processing, requires appropriately formulated ceramic pastes. These materials must combine suitable rheological parameters with the desired physicochemical properties after sintering [62]. Inadequate rheology will hinder proper extrusion through the printing nozzle, leading to structural discontinuities in overly viscous systems with excessive green density or, conversely, uncontrolled material oozing and dimensional inaccuracy when the paste is too fluid. Consequently, the proper selection of the paste composition is crucial to ensure process repeatability in 3D printing and to achieve the required mechanical performance of the final parts.

Comparative analyses of oxide ceramics processed by conventional and additive routes show that direct ink writing/robocasting (DIW) affords high green packing and tunable porosity but is constrained by feature resolution (100–300 µm) and sensitivity to rheology, drying-induced cracking, and sintering shrinkage [63,64,65,66]. Operationally, DIW functions within a narrow rheological window (yield stress, shear-thinning, thixotropy). Even small deviations promote nozzle clogging, die swell, and material spreading/collapse, degrading dimensional accuracy and surface finish [42,50,65]. The layer-wise filament deposition creates inter-filament and inter-layer interfaces where incomplete fusion and voids can arise, leading to microstructural anisotropy (build-direction-dependent properties) and visible surface terracing (“stair-stepping”) [42,63,64]. Drying of aqueous/solvent-based inks generates capillary stresses that drive warpage and cracking, while subsequent binder burnout and densification can amplify defect populations and cause substantial linear shrinkage requiring compensation at the design stage [42,63,64]. Additional practical constraints include limited self-supporting overhangs, a minimum printable wall thickness governed by nozzle/particle size [48,63]. Ink stability issues (sedimentation/agglomeration) further contribute to clogging and variability if formulation and processing are not tightly controlled [46,50]. Binder jetting reliably reproduces complex geometries under low thermal loads. However, it typically yields low green density, rough surfaces, and a resolution limited by powder size [67,68,69]. Ceramic stereolithography achieves the highest precision and near-full density, yet demands highly loaded, rheologically and photochemically stable slurries and necessitates support removal and shrinkage compensation at the design stage [70,71]. Material-specific limitations further govern performance: Y_2_O_3_-stabilized ZrO_2_ provides transformation toughening but is vulnerable to destabilization and deleterious tetragonal to monoclinic transformation if sintering/stabilization are not rigorously controlled [72,73], SiO_2_ may crystallize cristobalite during heat treatment, increasing shrinkage and embrittlement [74], TiO_2_ is prone to the anatase–rutile transformation and oxygen-defect sensitivity, requiring tight control of atmosphere and dwell time [75]. In this context, Al_2_O_3_ remains a chemically stable and cost-effective reference for benchmarking AM processes against conventional forming methods [63,64,65,66].

The present study investigates the competitiveness of 3D printing, specifically robocasting, as a manufacturing technology for oxide-ceramic components in comparison with conventional processing methods. The research focuses on evaluating the mechanical performance, microstructural characteristics, and material purity of oxide ceramics produced via robocasting. The primary objective is to establish quantitative relationships between processing parameters, resulting microstructure, and mechanical properties. A reproducible workflow encompassing paste formulation, printing, and sintering procedures was developed. The mechanical properties of the fabricated components were characterized through three-point bending, uniaxial compression, and hardness testing. The scanning electron microscopy (SEM) and X-ray diffraction (XRD) analyses were used for evaluating porosity and phase composition, with the measured values of strength, hardness, density, and Young’s modulus. Additionally, the study examines the spatial homogeneity of material properties across the printed components by integrating density measurements with spatially resolved hardness mapping. This approach enables the identification of potential anisotropies arising from the robocasting deposition path or layer-wise build sequence.

## 2. Materials and Methods

### 2.1. Sample Preparation

Foundry filters and rectangular prismatic specimens were fabricated by 3D printing robocasting, using a ceramic paste with the following composition: alumina powder (Al_2_O_3_; CT3000 SG, Almatis, Ludwigshafen, Germany), methylcellulose (E460, ITC, Piastów, Poland), dispersant (Darvan 7Ns, Vanderbilt Minerals LLC, Norwalk, CT, USA), and deionized water. The percentage composition of the ceramic mixture is listed in Table 1 [62].

The ceramic paste was prepared in a high-speed planetary centrifugal mixer (Thinky ARV-310, Thinky Corporation, Tokyo, Japan). The mixing protocol comprised three stages. In the first stage, all paste constituents were premixed at 750 rpm for 10 s. The rotational speed was then increased to 1100 rpm and mixing continued for 30 s. To ensure complete homogenization, a final stage at 2000 rpm was applied for 60 s.

**Figure 1:**
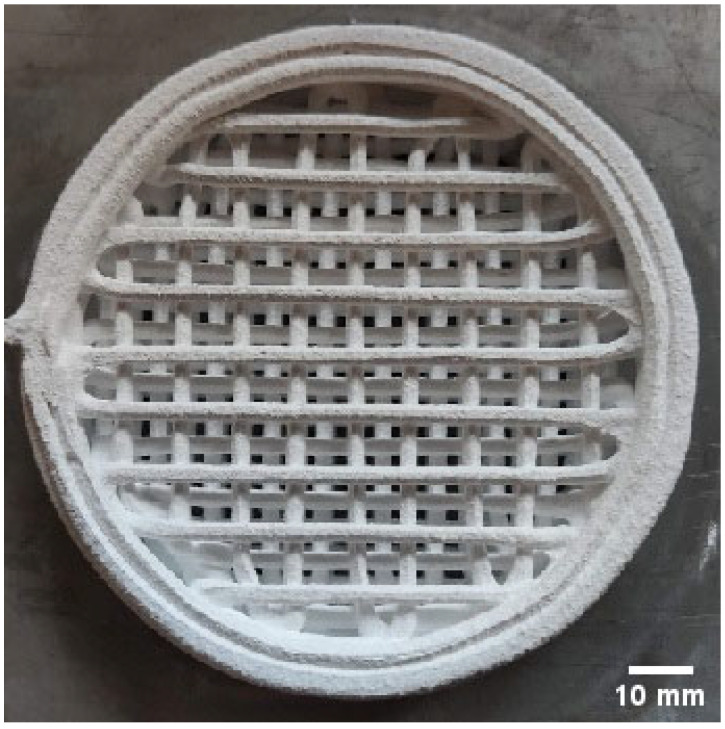
Representative view of the printed ceramic filter.

The prepared ceramic paste was subsequently used to fabricate ceramic filters (Figure 1) and rectangular prismatic specimens with a cross-section of 10 × 10 mm. The prismatic specimens were printed with solid infill (100%). During 3D printing, a 2 mm nozzle was employed.

**Figure 2:**
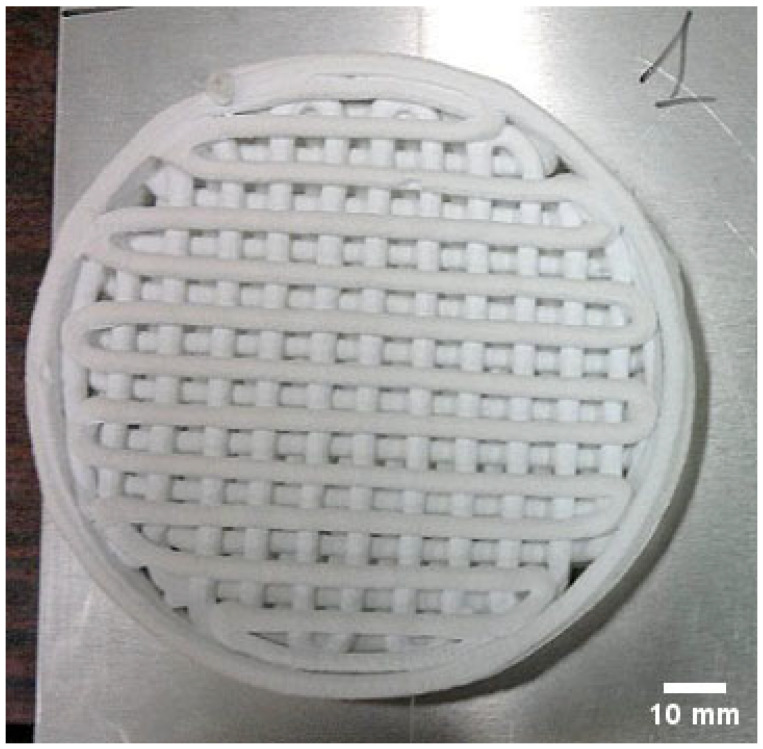
Representative view of the ceramic filter after thermal debinding—removal of organic constituents.

The next stage of the process comprised drying and removal of organic constituents (debinding). Drying was carried out under ambient conditions at 21 °C for 48 h. The dried filters and rectangular prismatic specimens were then subjected to a burnout step at 200 °C for 2 h, with a heating rate of 10 °C/h. A representative view of the filter after the organic-removal (debinding) stage is shown in Figure 2.

**Figure 3:**
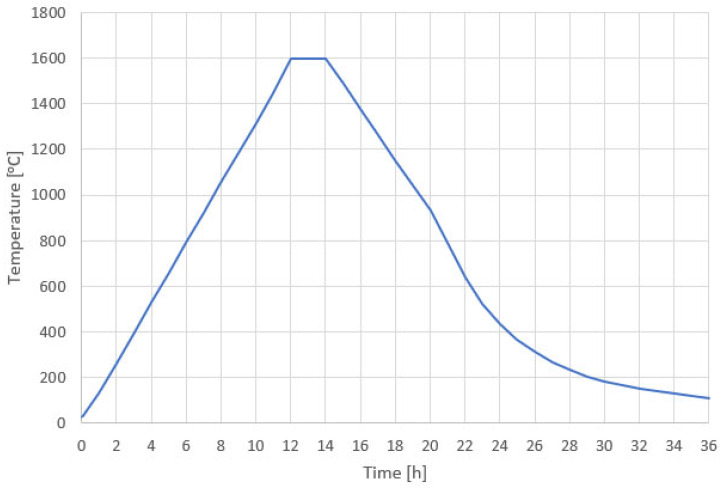
Sintering temperature profile for Al_2_O_3_ technical ceramics.

The final stage of the process was ceramic sintering. Samples were sintered in ambient air (no controlled atmosphere was employed). The furnace was heated at an average rate of ~133 °C/h to 1600 °C, soaked at 1600 °C for 2 h, and then furnace-cooled (cooled with the furnace) with an average cooling rate of ~68 °C/h between 1600 and 100 °C, followed by passive cooling to room temperature. The total thermal cycle time was ~36 h. The sintering temperature profile is shown in Figure 3.

### 2.2. Three-Point Bending Strength Testing

Flexural strength tests were performed using a testing machine with a maximum load capacity of 400 kN and a speed was set to 1 mm/min. The tests were conducted in accordance with PN-EN 843-1:2007 [76].

**Figure 4:**
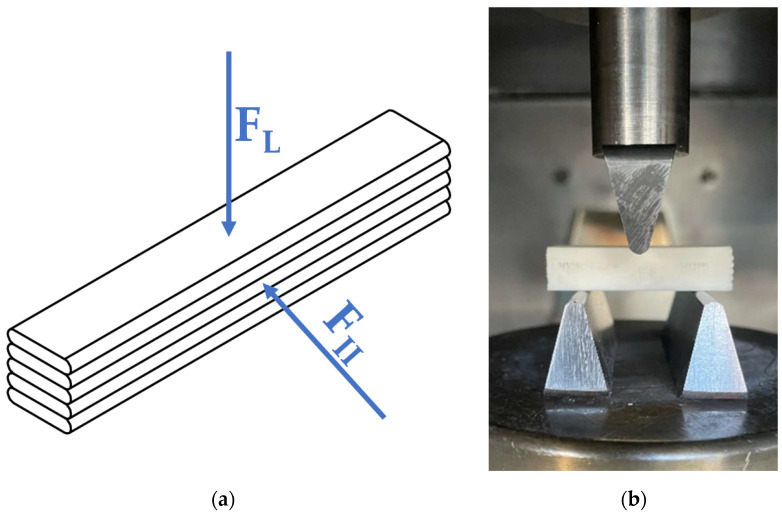
Three-point bending of solid specimens: (**a**) schematic of the loading direction relative to the printed layers (F_L_ for SBL samples and F_II_ for SBII samples); (**b**) representative view of a mounted specimen in the testing machine during the test.

Tests were carried out on solid rectangular prismatic specimens (10 × 10 × 60 mm), considering two loading directions with respect to the layer orientation resulting from the printing process. Specimens were designated SB (solid bar), with an additional suffix specifying the loading orientation: SBL for loading perpendicular to the layers, and SBII for loading parallel to the layers. The test was conducted at a speed of 1 mm/min. A schematic of the load orientation relative to the layers and an example of a mounted specimen during three-point bending are shown in Figure 4.

### 2.3. Compressive Strength Testing

**Figure 5:**
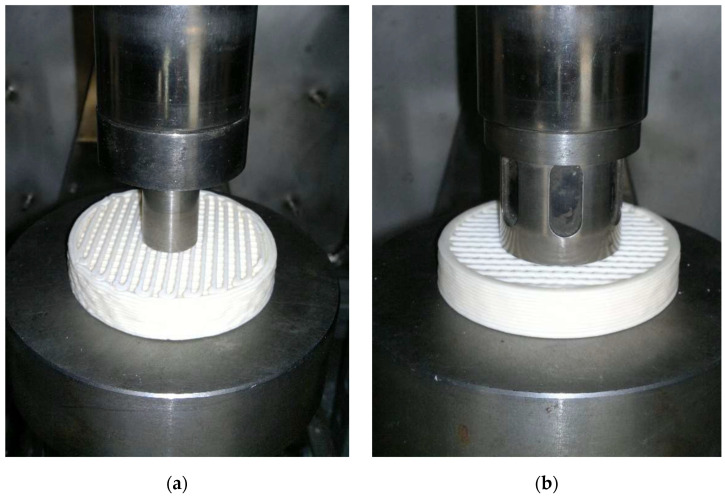
Representative view of ceramic filters during compressive strength testing: (**a**) 22 mm-diameter platen (F_P22_); (**b**) 40 mm-diameter platen (F_P40_).

Compressive strength tests were performed using a testing machine with a maximum load capacity of 400 kN, equipped with a mechanism for controlling the crosshead speed and fitted with compression platens of diameters D = 22 mm and D = 40 mm and a speed was set to 1 mm/min. The tests employed robocast solid cylindrical specimens with dimensions D = 11 mm, h = 11 mm and D = 15 mm, h = 15 mm, designated SC (solid cylinder) ceramic filters designated as F_P22_ for a platen diameter of 22 mm and F_P40_ for a platen diameter of 40 mm were used for the tests. Representative images of the filters prepared for bending tests are shown in Figure 5.

### 2.4. Microstructural Analysis

**Figure 6:**
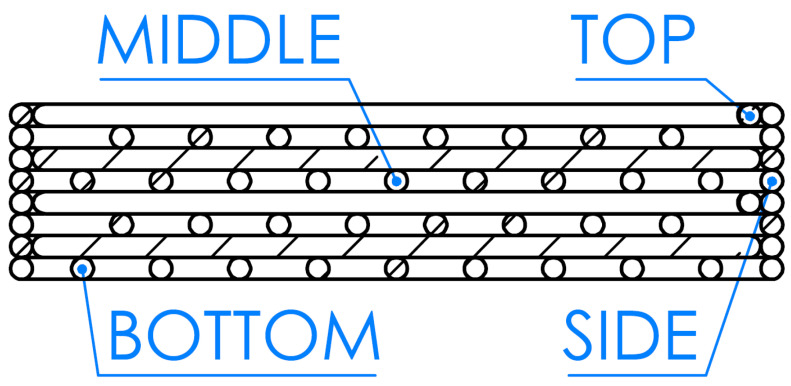
Schematic of the locations for microscopic observations and hardness measurement points on the metallographic cross-sections of specimens sampled from the ceramic filters.

Selected areas of metallographic cross-sections were subjected to microstructural analysis using scanning electron microscopy (SEM; JSM-6460LV, JEOL, Tokyo, Japan). Observations were carried out for:

- two solid specimens formed by robocasting and pressureless sintered, designated SC (solid cylinder);
- five ceramic filters, examined at different locations across the transverse cross-section—top (FT), middle (FM), bottom (FB), and side (FS); see Figure 6—in backscattered electron (BSE) and secondary electron (SEI) modes, at an accelerating voltage of 10 kV and magnifications in the range of 50–1000×. In addition, macrographs of selected filter regions were acquired at 50× in SEI mode.

**Figure A1:**
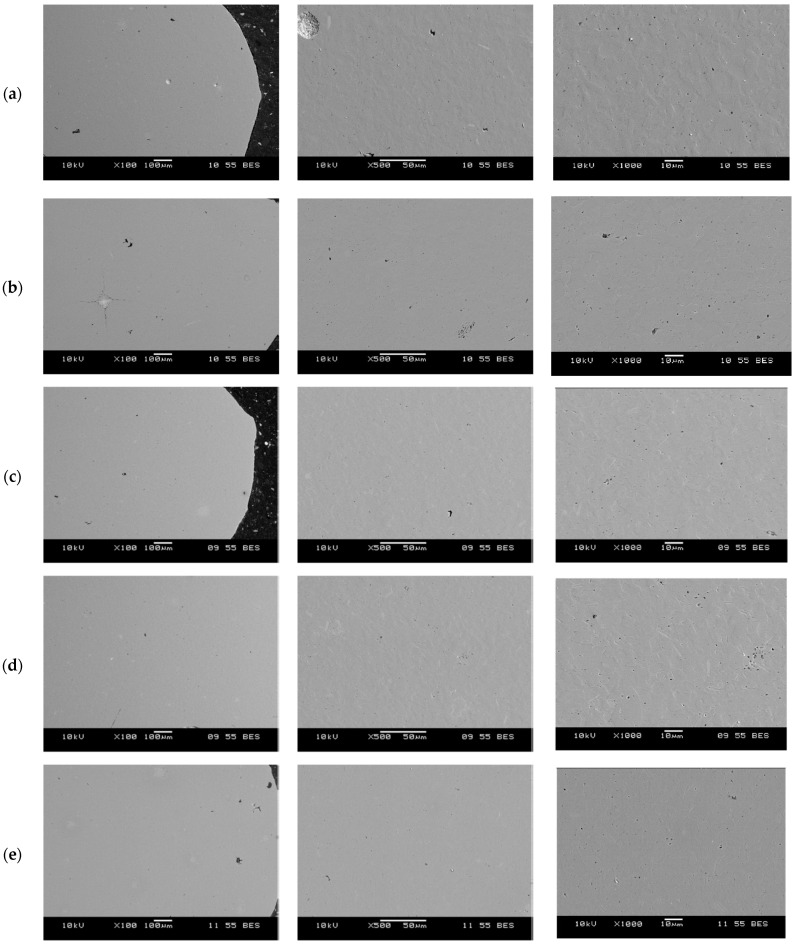
Microstructures of the five samples of filters in the top lamella regions (FT) at magnifications of 100×, 500×, and 1000× (BSE mode): (**a**) Sample 1, (**b**) Sample 2, (**c**) Sample 3, (**d**) Sample 4, (**e**) Sample 5.

**Figure A2:**
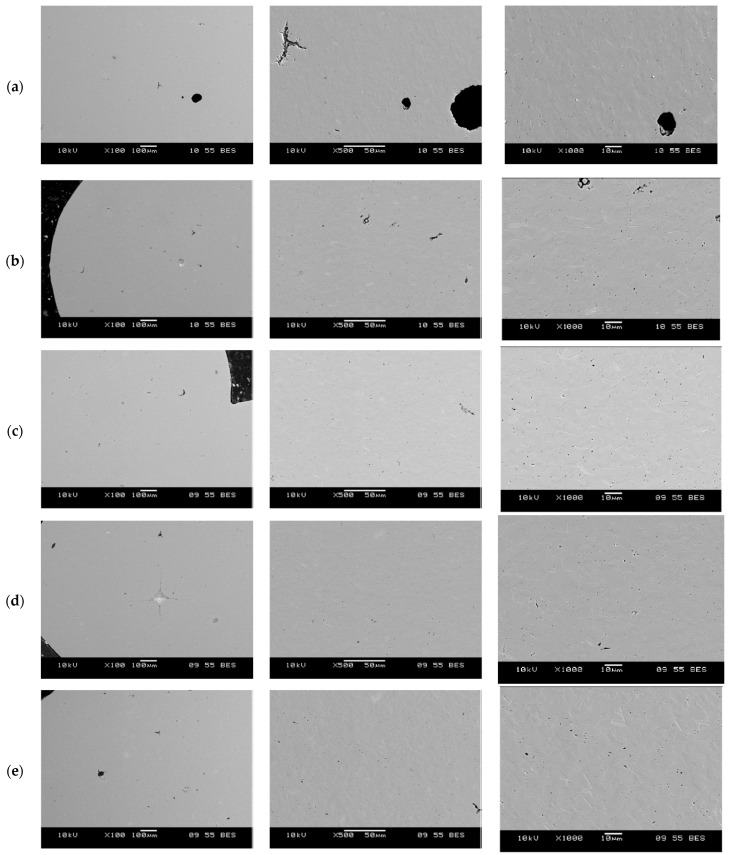
Microstructures of the five samples of filters in the middle lamella regions (FM) at magnifications of 100×, 500×, and 1000× (BSE mode): (**a**) Sample 1, (**b**) Sample 2, (**c**) Sample 3, (**d**) Sample 4, (**e**) Sample 5.

**Figure A3:**
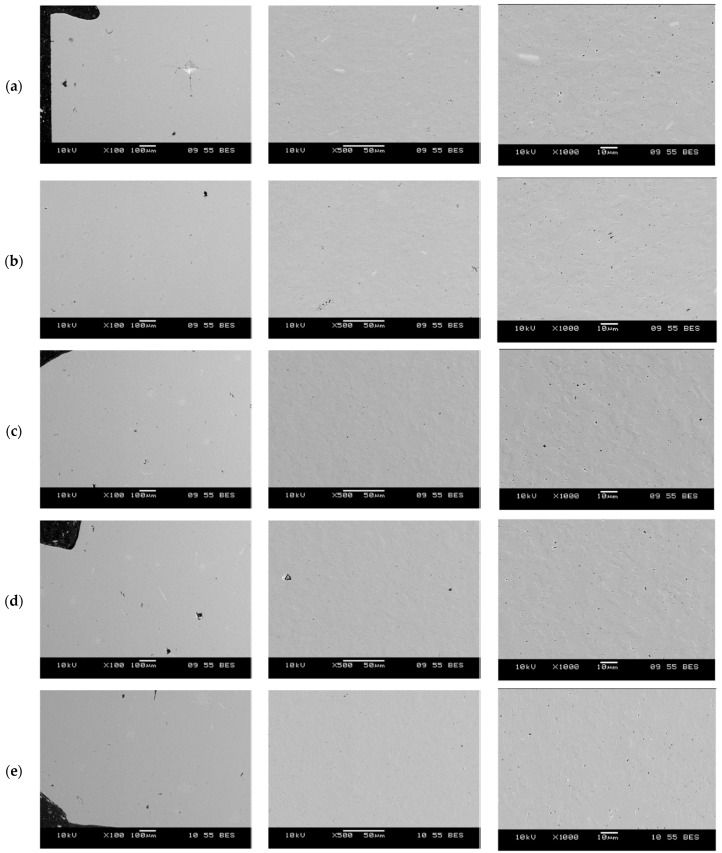
Microstructures of the five samples of filters in the bottom lamella regions (FB) at magnifications of 100×, 500×, and 1000× (BSE mode): (**a**) Sample 1, (**b**) Sample 2, (**c**) Sample 3, (**d**) Sample 4, (**e**) Sample 5.

**Figure A4:**
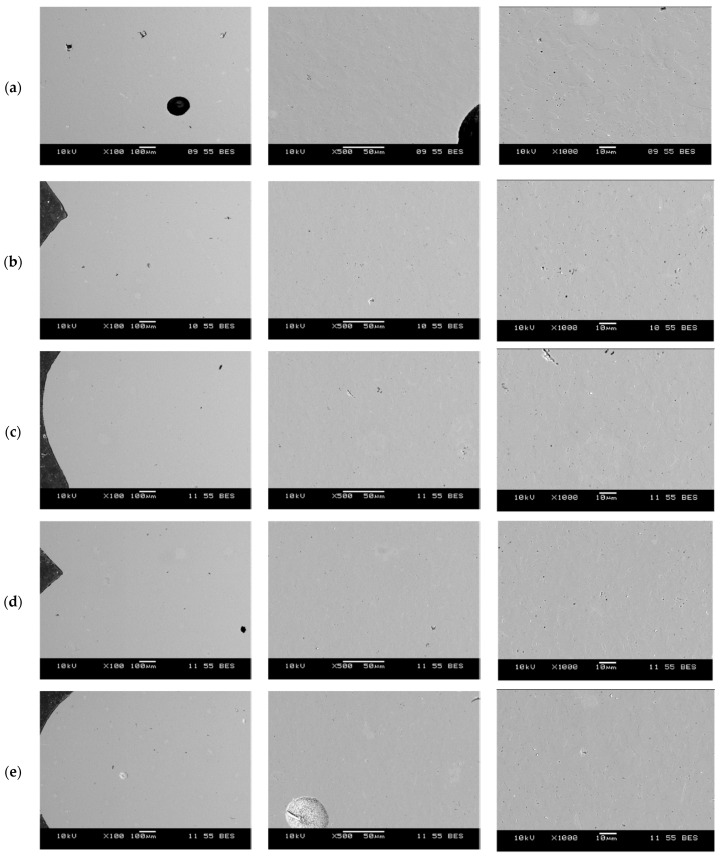
Microstructures of the five samples of filters in the external wall regions (FS) at magnifications of 100×, 500×, and 1000× (BSE mode): (**a**) Sample 1, (**b**) Sample 2, (**c**) Sample 3, (**d**) Sample 4, (**e**) Sample 5..

A compilation of all micrographs at 100×, 500×, and 1000× is provided in Appendix A. The compilations are grouped by the respective filter regions: FT (Figure A1), FM (Figure A2), FB (Figure A3), and FS (Figure A4).

### 2.5. Hardness Testing

Hardness testing of specimens cut from the ceramic filter structure was performed by the Vickers method in accordance with PN-EN 843-4:2006 [77]. Measurements were carried out using a digital Vickers hardness tester FLC-50VX (FUTURE-TECH, Kawasaki, Japan) with applied loads of 9.81 N (HV1) and 98.1 N (HV10).

The analysis was conducted on metallographic cross-sections prepared from transverse sections of specimens cut from the ceramic filters and, for comparison, on solid specimens (SC) after pressureless sintering. Following the adopted methodology, measurements were taken in four characteristic regions of the filters: the top lamella (FT), middle lamella (FM), bottom lamella (FB), and the side wall (FS), as schematically shown in Figure 6.

Fracture toughness was determined by the indentation method, i.e., by evaluating the critical stress intensity factor KIC(HV). The procedure involves applying a load to produce a Vickers indentation from whose corners cracks initiate, followed by measuring the indentation diagonals and the lengths of the resulting cracks. The Niihara method was used for the calculations.

**Figure 7:**
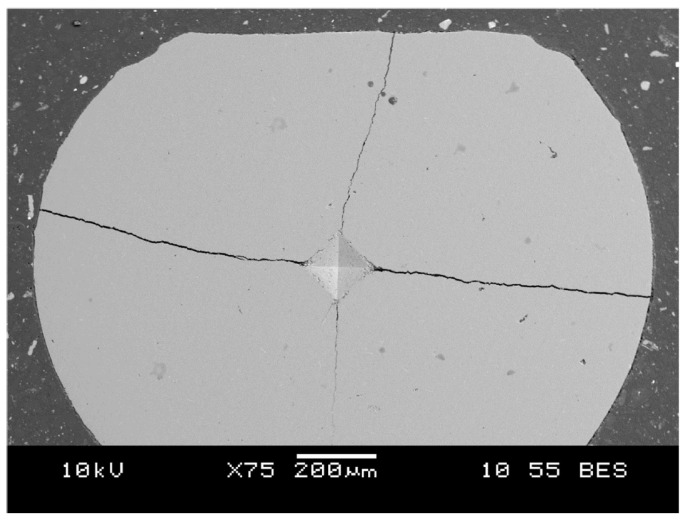
Representative view of the Vickers indentation after applying a 294.2 N load (HV30) on the filter cross-section in the top lamella region (FT).

An attempt was made to determine fracture toughness at a load of 30 kgf (HV30); however, the generated cracks reached the edges of the lamella cross-sections (Figure 7), which precluded a reliable determination of KIC(HV). Consequently, a load of 10 kgf (98.1 N; HV10) was employed, which yielded indentations with proper geometry and single cracks emanating from the indentation corners. Based on the measured indentation diagonals and crack lengths, the values of KIC(HV) were calculated.

### 2.6. Phase Composition Analysis

The surfaces of the sintered solid cylindrical (SC) specimens were examined by X-ray diffraction (XRD) using an Empyrean diffractometer (PANalytical, Worcestershire, UK) equipped with a copper-anode X-ray tube (Cu Kα_1_ = 1.5419 Å), a nickel filter, and a PIXcel3D detector. Data acquisition and analysis were performed with PANalytical HighScore Plus software version 4.8 integrated with the ICDD PDF-4+ 2023 crystallographic database. Phase analysis was conducted on diffractograms recorded in Bragg–Brentano geometry.

### 2.7. Density Determination

The materials were subjected to apparent-density measurements in accordance with PN-EN 623-2:2001 [78]. The measurement was performed on a PS 1000.R2 precision scale (Radwag, Radom, Poland) with a RADWAG KIT 128 accessory. The apparent density *ρ_o_* was calculated using Equation (1):

(1) $$\rho_{o}=\frac{m_{1}}{m_{3}-m_{2}}\cdot \rho_{l}$$

where

*ρ_o_*—apparent density of the material [g/cm^3^],
*m*_1_—dry mass of the specimen [g],
*m*_2_—apparent (buoyant) mass of the immersed specimen in the liquid [g],
*m*_3_—mass of the specimen saturated with the liquid, [g],
*ρ_l_*—density of the liquid used for weighing [g/cm^3^].

### 2.8. Young’s Modulus Determination

Young’s modulus was determined by the ultrasonic method. Transit times of longitudinal and transverse ultrasonic waves through the specimens were measured using a digital flaw detector EPOCH 3 (Olympus, Tokyo, Japan) equipped with broadband transducers. The elastic constants were calculated from the ultrasonic wave velocities in the material and the specimen density using the Modulus 1.0 software. Young’s modulus was then computed using the following Equation (2):

(2) $$E=p\cdot C_{T}^{2}\frac{3C_{L}^{2}-4C_{T}^{2}}{C_{L}^{2}-C_{T}^{2}}$$

where

*E*—Young’s modulus [GPa],
*C_L_*—longitudinal wave velocity [km/s],
*C_T_*—transverse (shear) wave velocity [km/s],
*p*—material density [g/cm^3^].

## 3. Results

### 3.1. Phase Composition Analysis

**Figure 8:**
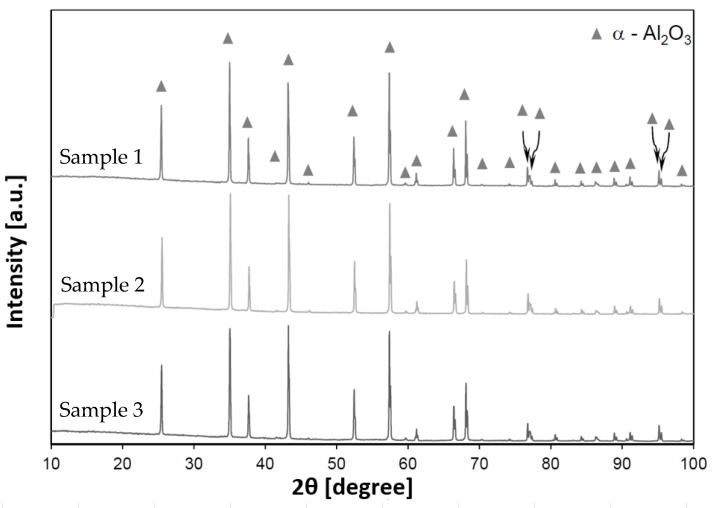
Representative X-ray diffractogram of the SC sample after sintering.

The results of the phase composition analysis of the sintered materials shaped as solid cylinders (SC) are presented in Figure 8.

Phase composition analysis confirmed the presence of α-phase alumina (Al_2_O_3_, card no. 00-046-1212), with no other crystalline phases detected.

### 3.2. Young’s Modulus and Density Determination

Apparent density, bulk density, porosity, and Young’s modulus were measured on SC (solid cylinder) samples. The results indicate that cylinders formed from the ceramic paste—comprising predominantly alumina (Al_2_O_3_) with organic additives removed in subsequent firing steps—exhibit physical properties comparable to technical ceramics produced by conventional routes [79,80,81]. The measured mean apparent density for specimens SC1–SC5 was 3.91 g/cm^3^, with an average open porosity of 0.34% and an average Young’s modulus of 384.4 GPa. Detailed results of apparent and bulk density measurements and Young’s modulus, together with a benchmark for conventional manufacturing technologies, are summarized in Table 2.

### 3.3. Microstructural Analysis

**Figure 9:**
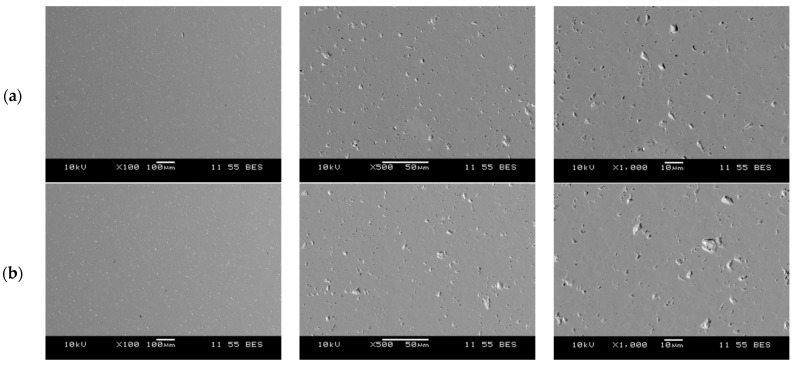
Microstructure of solid specimens formed by robocasting and pressureless-sintered: (**a**) SC1; (**b**) SC2.

Microstructural observations performed by scanning electron microscopy (SEM) showed that, irrespective of specimen geometry—both solid cylinder samples (SC) and ceramic filters (FT, FM, FB, FS)—the material exhibits a high degree of densification. In the microstructures of the solid specimens (Figure 9), no cracks, unbonded material, or significant porosity were observed; the size of individual closed pores did not exceed 10 µm.

**Figure 10:**
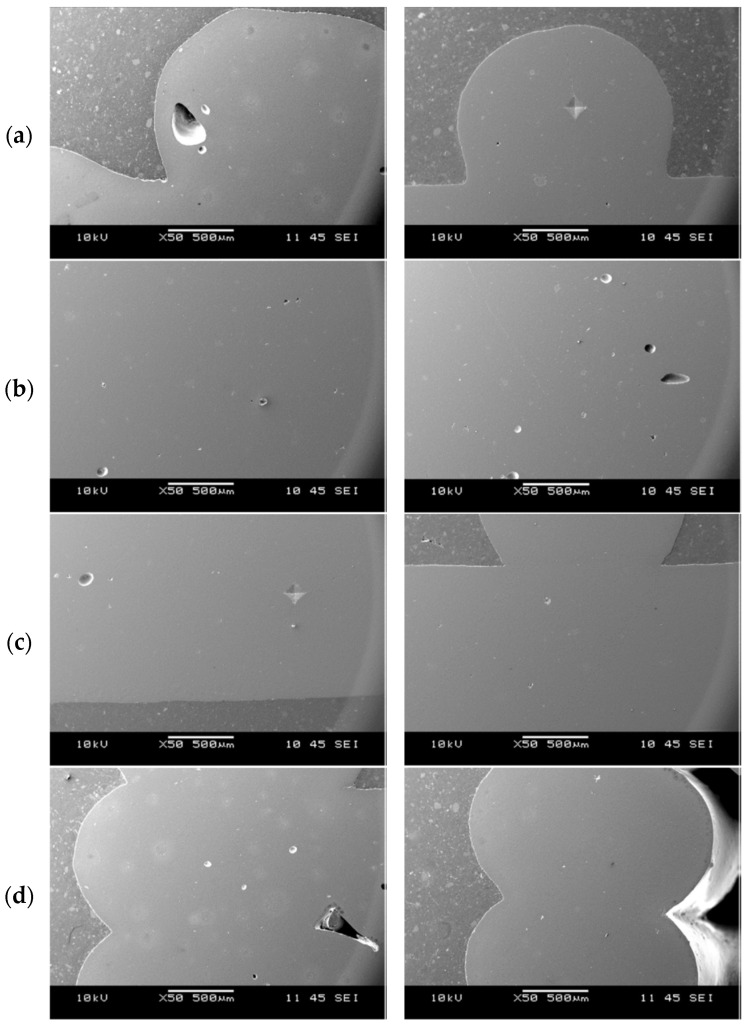
Representative microstructures of the filters from the regions: (**a**) FT, (**b**) FM, (**c**) FB, (**d**) FS.

For the ceramic filters (Figure 10), analysis conducted in all four inspection regions (Figure 6) revealed the presence of minor structural discontinuities in the form of closed pores, which may arise from incomplete degassing of the ceramic paste prior to printing. Furthermore, the interface between successive transverse layers is continuous. This is important because, in this zone, adjacent material layers meet at 90°, and the interface width corresponds to the diameter of the nozzle used in the 3D-printing process. Such regions may serve as sites for the accumulation of residual stresses generated by material shrinkage during drying and sintering. It should also be noted that the cross-section of an individual printed layer is approximately circular, which can lead to local notch formation at layer junctions (Figure 10d). These discontinuities may constitute potential sites for crack initiation and propagation under mechanical and thermal loading.

### 3.4. Hardness Testing

**Figure 11:**
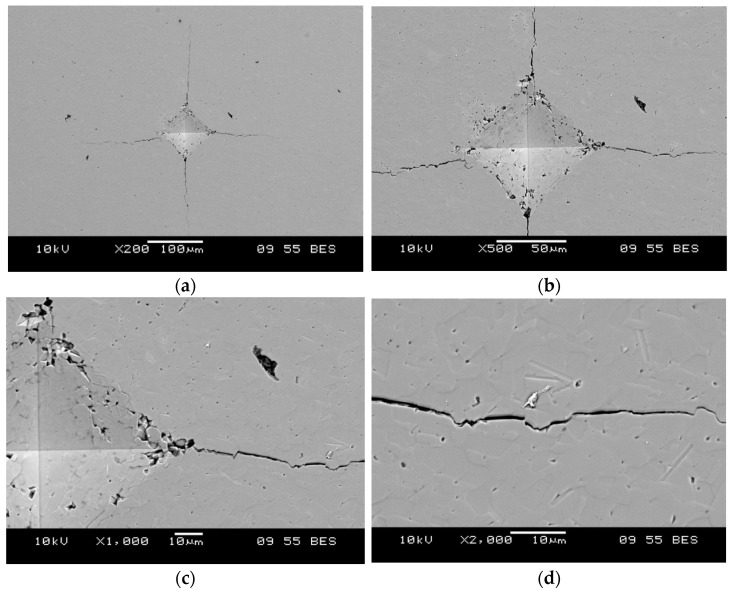
Representative microstructures with a Vickers indentation and associated cracks after applying a 98.1 N load (HV10) at magnifications: (**a**) 200×, (**b**) 500×, (**c**) 1000×, (**d**) 2000×.

Hardness and fracture toughness measurements were performed both on pressureless-sintered solid materials and on ceramic filters in the top, middle, and bottom lamella regions, as well as within the side walls. Microstructures showing Vickers indents (HV10) with the associated cracks are presented in Figure 11.

Vickers hardness measurements (HV10) showed that both solid materials and the printed filters in all analyzed regions (FT, FM, FB, FS) exhibit hardness values in the range 16.9–17.6 GPa, confirming the absence of significant differences between the specimen preparation routes and among the respective zones. An analogous trend was observed under the higher load (98.1 N, HV10), for which the hardness values were 15.3–15.6 GPa. For comparison, classical manufacturing methods—slip casting, ceramic injection molding (CIM), and dry pressing—are reported to yield hardness values of 14.7, 19.61, and 14.8 GPa, respectively [79,80,83].

The critical stress intensity factor KIC was determined for both the solid materials and the printed filters, with measurements conducted in the four regions (FT, FM, FB, FS). Regardless of specimen preparation route and measurement location, the obtained values lay within 3.46–3.64 MPa·m^1/2^. Comparing the mechanical properties of the filters with those of the pressureless-sintered solid printed specimens, it is evident that the mean HV1, HV10, and KIC (HV) values for the filters are similar, showing only slightly lower values than those of the solid materials. Detailed results of the hardness and critical stress intensity factor measurements are summarized in Table 3.

### 3.5. Three-Point Bending Strength Testing

Table 4 presents the results of three-point flexural strength tests for rectangular specimens fabricated by robocasting. Specimens loaded perpendicular to the printed-layer direction (SBL) attained bending forces in the range 4.2–6.4 kN, corresponding to stresses of 378–576 MPa. In contrast, specimens loaded parallel to the layer-deposition direction (SBII) exhibited maximum forces of 5.6–5.8 kN, translating into stresses of 504–522 MPa. Moreover, greater result repeatability was observed for SBII specimens than for SBL sample.

For comparison, the literature and industrial practice commonly report that for technical-grade alumina (95–97% Al_2_O_3_) the flexural strength lies in the range 200–350 MPa, whereas for high-purity ceramics (99.5–99.9% Al_2_O_3_) the values are 300–450 MPa. The present results therefore indicate that the robocast samples exhibit higher flexural strength than ceramics produced by standard manufacturing routes, which are 418 MPa for slip casting, 250–350 MPa for CIM, and 300–500 MPa for dry pressing [81,84,85].

**Figure 12:**
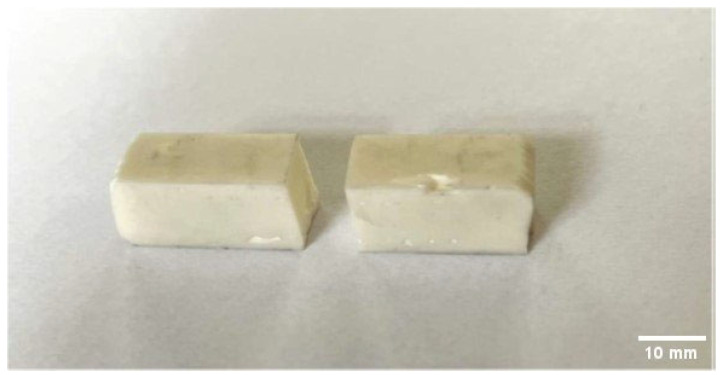
Representative view of the solid bar sample fracture surface after the three-point bending test, with the load applied parallel to the direction of successive layer deposition in the robocasting printing process.

Figure 12 shows the fracture edge of a rectangular specimen subjected to a bending load applied perpendicular to the printed-layer direction. The fracture plane is straight and exhibits no ragged edges, indicating a dominant brittle fracture mechanism—typical of ceramics—in which the absence of plastic deformation prevents energy dissipation in the fracture process zone. Consequently, crack initiation occurs at local microstructural defects (e.g., micropores or discontinuities), and subsequent propagation proceeds rapidly along the plane of lowest resistance to crack growth.

### 3.6. Compressive Strength Testing

**Figure 13:**
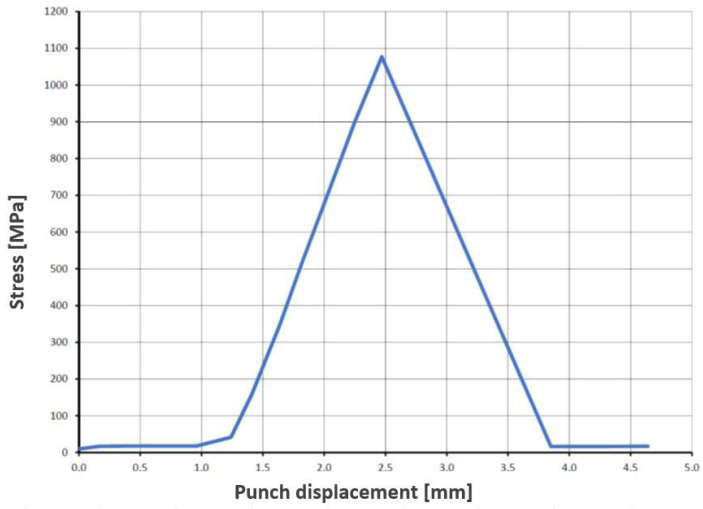
Representative load–displacement curve recorded during compression testing of a solid cylinder sample, fabricated by robocasting.

During compressive strength analyses of solid specimens fabricated by robocasting, it was observed that the applied load increases linearly up to the compressive strength limit. Once this limit is exceeded, catastrophic failure occurs, resulting in an abrupt drop in the recorded load. A representative load–displacement curve is shown in Figure 13. Such a response is characteristic of brittle ceramic materials.

The results of compressive strength tests for solid cylinder (SC) samples are summarized in Table 5. For cylinders with a diameter of 11 mm and a height of 11 mm (SC1–SC6), the maximum compressive load ranged from 55.6 to 163.3 kN, corresponding to compressive strengths from 585.1 MPa (SC4) to 1718.3 MPa (SC5). A pronounced scatter in the results is evident, which may indicate the presence of internal defects—structural discontinuities (voids)—in specimens with lower maximum load values (<120 kN, i.e., SC1 and SC4). For cylinders with a diameter of 15 mm and a height of 15 mm (SC7–SC12), the maximum compressive load was in the range 190.3–320.0 kN, with corresponding compressive strengths from 1076.9 MPa (SC7) to 1810.8 MPa (SC9).

**Figure 14:**
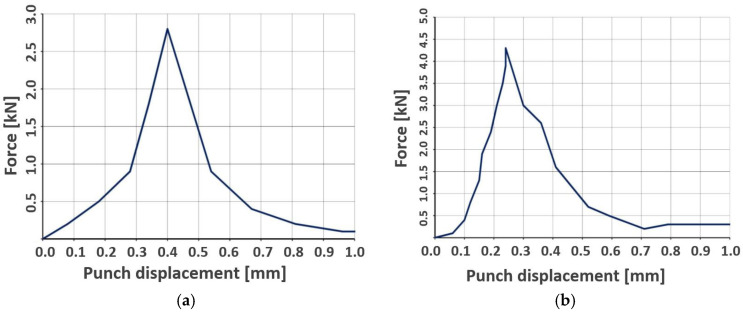
Force–punch displacement curve recorded during filter bending: (**a**) 22 mm-diameter loading platen; (**b**) 40 mm-diameter loading platen.

Compared with the solid specimens, the curves illustrating the compressive load measurement for the ceramic filters (Figure 14) exhibit a more stepwise (staircase-like) evolution of the recorded load, particularly when using the 40 mm-diameter platen.

The compressive force required to fracture the ceramic filters when loaded with the 40 mm-diameter platen ranged from 2.6 to 4.3 kN. For the 22 mm-diameter platen, the maximum values were 2.2 to 4.0 kN. These ranges are similar, which follows from the lattice architecture of the ceramic filters: this structure yields only a slight increase in the effective cross-sectional area beneath the larger platen, thereby limiting differences in the transmitted force. It should be emphasized that, due to the regular, porous geometry of the filters, the stress distribution under load is non-uniform, and failure initiates in local stress-concentration zones—most often at lattice rib junctions. The fracture mechanism thus differs from that observed in solid materials, where failure proceeds more uniformly and globally. A summary of the filter bending test results is presented in Table 6.

**Figure 15:**
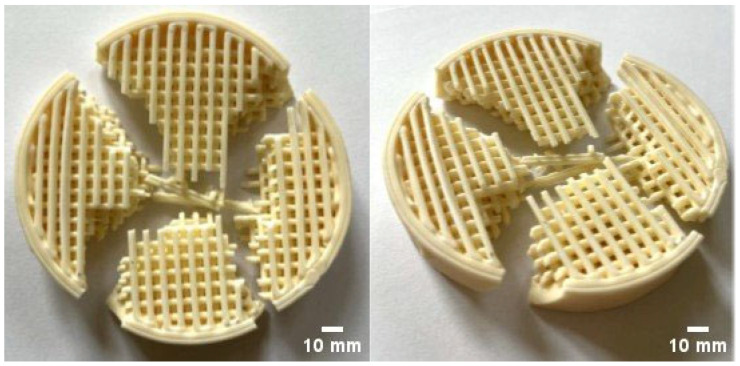
Representative view of the ceramic filter after compression with the 40 mm-diameter platen.

Figure 15 shows the ceramic filter after the compression test. It was observed that the crack traversed the entire volume of the specimen, propagating from the central region toward the lateral edge. As a result, the filter fractured into four relatively equal fragments. The crack-propagation pattern is associated with the lattice architecture of the filter, in which successive material layers are arranged at 90° to one another.

## 4. Discussion

Microstructural, phase-composition, and mechanical tests were carried out on ceramic materials fabricated by robocasting. Phase analysis confirmed the presence of alumina (Al_2_O_3_) in the α modification, with no other phases detected.

Microstructural examination of the specimens and filters produced by robocasting revealed grain sizes below 10 μm. The material exhibited a continuous structure, with pores not exceeding 10 μm. Particular attention should be paid to the interlayer interfaces. Additive technologies, by virtue of their layer-wise build strategy, are prone to issues of structural continuity in these regions. This is especially critical for ceramic materials, which require subsequent thermal treatments. Interfacial discontinuities can serve as potential sites for crack nucleation and propagation during later processing steps such as drying, burnout, or sintering. Moreover, if the printed component is intended to operate at elevated temperatures, any structural imperfections will adversely affect fracture toughness and thermal-shock resistance. It is also worth noting that a circular nozzle cross-section may not be optimal, as notches can form at both transverse and longitudinal layer junctions—features that can act as easy crack-initiation sites. In particular, for perpendicular layer interfaces, the notch exhibits a sharp transition from one layer to the next.

The density of materials manufactured by this method ranged from 3.88 to 3.93 g/cm^3^. These materials showed low open porosity—below 0.6%—and a Young’s modulus of approximately 380 GPa. The solid ceramic specimens exhibited high hardness, HV1 ≈ 18 GPa (HV10 ≈ 16 GPa), and a fracture toughness of 3.80 MPa·m^1/2^. Hardness and fracture-toughness measurements were also performed for the ceramic filters (on the side surface and on the top, middle, and bottom lamellae) printed by robocasting. The hardness and fracture toughness of the respective filter regions were comparable to those measured for the solid specimens. The filters’ HV1 hardness lay in the range 16.7–18.0 GPa (HV10 = 14.8–16.1 GPa), while their fracture toughness ranged from 3.40 to 3.66 MPa·m^1/2^.

A substantial scatter in the mechanical properties of robocast materials was observed, which may be influenced by local structural discontinuities. The flexural strength of specimens produced by this method ranged from 200 to 580 MPa, and the compressive strength from 1000 to 1800 MPa. Bending tests of the ceramic filters were also performed using punches of D = 22 mm and D = 40 mm diameter. Regardless of the punch used, the filters withstood applied forces in the range 2.2–4.6 kN. This follows from the specific lattice architecture of the filters, in which increasing the nominal contact area under a larger punch does not produce a commensurate increase in the effective load-bearing cross-section.

## 5. Conclusions

Based on the conducted investigations, technical ceramics manufactured by the modern additive process of robocasting exhibit mechanical performance comparable to oxide ceramics produced by more conventional routes such as slip casting, ceramic injection molding (CIM), and dry pressing. Moreover, the quantified pore content is markedly lower than in the case of conventional methods.

From the chemical composition analysis of the sintered ceramic components, it can be inferred that the necessary additives used to formulate the ceramic paste—the carrier for the ceramic phase in the 3D-printing process—do not introduce deleterious contamination into the final product.

Microstructural analysis confirmed that the successive layers deposited during robocasting exhibit structural continuity at the interlayer interfaces, which supports further development of oxide–ceramic processing by this technology.

In addition, the mechanical and materials evaluations of the printed ceramic filters indicate that 3D printing robocasting in particular can serve as a viable alternative manufacturing route, enabling the fabrication of components with more complex architectures than those attainable by currently employed production methods, while maintaining the required mechanical and material-property targets.

## Figures and Tables

**Table 1:** Chemical composition of ceramic paste adapted for 3D printing using robocasting technology.

Material	Chemical Composition [wt.%]
Al_2_O_3_	78.5
H_2_O	21.15
Dispersant	0.1
Methylcellulose	0.25

**Table 2:** Compilation of apparent and bulk density measurements and Young’s modulus for ceramic sinters fabricated by robocasting.

Sample	Apparent Density *ρ_o_* [g/cm^3^]	Mean Apparent Density *ρ_o_* [g/cm^3^]	Open Porosity [%]	Mean Open Porosity [%]	Young’s Modulus E [GPa]	Mean Young’s Modulus E [GPa]
SC1	3.88	3.91 ± 0.02	0.6	0.34 ± 0.23	380	384.4 ± 2.7
SC2	3.91		0.5		386	
SC3	3.91		0.4		384	
SC4	3.93		0.1		385	
SC5	3.93		0.1		387	
Slip Casting [82,83]	3.86		3		370–385	
Ceramic Injection Molding (CIM) [82,84]	3.58–3.96		2–8		360–385	
Dry Pressing [82]	3.85–3.90		1–3		370–400	

**Table 3:** Results of hardness and fracture toughness measurements for solid cylinder samples (SC) and filters as a function of the measurement location (FT, FM, FB, FS).

Sample	HV1 [GPa]	Mean HV1 [GPa]	HV10 [GPa]	Mean HV10 [GPa]	K_IC_(HV) [MPa∙m^1/2^]	Mean K_IC_(HV) [MPa∙m^1/2^]
SC1	18.2	17.8 ± 0.44	16.5	16.2 ± 0.38	3.82	3.80 ± 0.03
SC2	17.2		15.5		3.78	
SC3	17.4		17.0		3.77	
SC4	17.9		16.1		3.84	
SC5	18.1		15.7		3.81	
FT1	17.2	17.0 ± 0.18	15.8	15.7 ± 0.14	3.51	3.47 ± 0.04
FT2	17.0		15.8		3.47	
FT3	17.0		15.2		3.44	
FT4	16.9		16.1		3.51	
FT5	16.7		15.6		3.40	
FM1	17.5	17.6 ± 0.28	15.6	15.4 ± 0.18	3.42	3.46 ± 0.05
FM2	17.4		15.5		3.52	
FM3	18.0		15.4		3.40	
FM4	17.3		15.2		3.46	
FM5	17.6		15.4		3.50	
FB1	17.4	16.9 ± 0.27	15.3	15.6 ± 0.16	3.56	3.60 ± 0.03
FB2	17.0		15.6		3.63	
FB3	16.8		15.6		3.59	
FB4	16.8		15.7		3.62	
FB5	16.7		15.7		3.60	
FS1	17.5	17.1 ± 0.30	14.8	15.3 ± 0.25	3.69	3.64 ± 0.04
FS2	16.7		15.5		3.58	
FS3	17.3		15.8		3.66	
FS4	17.1		15.2		3.60	
FS5	17.2		15.2		3.68	

**Table 4:** Three-point flexural strength of rectangular specimens as a function of the loading direction relative to the direction of successive layer deposition in the printing process.

Sample	Dimensions		Force F [kN]	Three-Point Flexural Strength G [MPa]	Mean Three-Point Flexural Strength G [MPa]
	b [mm]	h [mm]			
SBL1	10	10	6.4	576	480 ± 99
SBL2			4.2	378	
SBL3			5.4	486	
SBII1			5.6	504	516 ± 10
SBII2			5.8	522	
SBII3			5.8	522	

**Table 5:** Compressive strength of solid cylinder (SC) samples fabricated by robocasting.

Sample	Dimensions		Compressive Force F [kN]	Compressive Strength G [MPa]	Mean Compressive Strength G [MPa]
	D [mm]	h [mm]			
SC1	11	11	63.5	668.2	1205.53 ± 475.33
SC2			152.2	1601.5	
SC3			131.3	1381.6	
SC4			55.6	585.1	
SC5			163.3	1718.3	
SC6			121.5	1278.5	
SC7	15	15	190.3	1076.9	1379.15 ± 280.71
SC8			269.0	1522.2	
SC9			320.0	1810.8	
SC10			233.0	1318.5	
SC11			192.0	1086.5	
SC12			258.0	1460.0	

**Table 6:** Compressive force recorded during testing of ceramic filters fabricated by robocasting.

Sample	Platen Diameter D [mm]	Compressive Force F [kN]	Mean Compressive Force F [kN]
F1_P40_	40	4.3	3.40 ± 0.78
F2_P40_		2.6	
F3_P40_		4.1	
F4_P40_		2.7	
F5_P40_		3.3	
F6_P22_	22	2.8	2.94 ± 0.70
F7_P22_		3.2	
F8_P22_		4.0	
F9_P22_		2.5	
F10_P22_		2.2	

## Data Availability

The original contributions presented in this study are included in the article. Further inquiries can be directed to the corresponding author.

## Abbreviations

DIW	direct ink writing
Al_2_O_3_	aluminum oxide
ZrO_2_	zirconium oxide
Y_2_O_3_	yttrium oxide
SiO_2_	silicon dioxide
TiO_2_	titanium dioxide
AM	additive manufacturing
BJ	binder jetting
SLA	stereolithography
SEM	scanning electron microscopy
XRD	X-ray diffraction
SB	solid bar
SBL	solid par perpendicular force
SBII	solid bar parallel force
FT	filter top
FM	filter middle
FB	filter bottom
FS	filter side
BSE	backscattered-electron imaging
SEI	secondary-electron imaging
CIM	ceramic injection molding
F_P40_	filter platen (40 mm)
F_P22_	filter platen (22 mm)
